# Choroidal thickness changes in post-COVID-19 cases

**DOI:** 10.5935/0004-2749.20230021

**Published:** 2022-02-18

**Authors:** Şerife Gülhan Konuk, Rasit Kılıç, Bilge Türkyılmaz, Emine Türkoğlu

**Affiliations:** 1 Department of Ophthalmology, Tokat Gaziosmanpaşa University, Medical Faculty, Tokat, Turkey; 2 Department of Infectious Diseases and Clinical Microbiology Tokat Gaziosmanpaşa University Medical Faculty, Tokat, Turkey

**Keywords:** COVID-19, Coronavirus infections, Optical coherence tomography, Choroid/pathology, Thickness; Eye manifestations, COVID-19, Infecções por coronavirus, Tomografia de coerência óptica, Coróide/patologia, Manifestações oculares

## Abstract

**Purpose:**

The current study aimed to evaluate the effects of the coronavirus disease 2019 (COVID-19) on choroidal thickness using enhanced depth imaging optical coherence tomography.

**Methods:**

This study evaluated the right eyes from 41 post-COVID-19 cases (Group 1) and 41 healthy subjects (Group 2). Choroidal thickness was measured using enhanced depth imaging optical coherence tomography. Post-COVID-19 cases were evaluated within 1 month after a diagnosis of COVID-19. Two experienced ophthalmologists measured the choroidal thickness at the subfovea, temporal, and nasal quadrants at 500-µm intervals up to 1500 µm from the fovea at seven different points. Moreover, central macular thickness and ganglion cell layer thickness were measured via OCT, after which both two groups were compared.

**Results:**

Group 1 showed a significantly thicker choroid compared to Group 2 at the subfovea, 500 µm temporal to the fovea, 500 and 1000 µm nasal to the fovea (p=0.011, p=0.043, p=0.009, and p=0.019, respectively). Although other areas measured were also thicker in Group 1, the difference was not significant (p>0.05). Moreover, no significant difference in the central macular thickness and ganglion cell layer thickness were observed between the groups (p>0.05).

**Conclusion:**

Choroidal thickness was increased in post-COVID-19 patients, which might be related to inflammation associated with the pathogenesis of COVID-19.

## Abstract (pt)

**Objetivo:**

Avaliar o efeito da doença por coronavírus de 2019 (COVID19) na espessura da coroide usando tomografia de coerência óptica com profundidade de imagem aprimorada.

**Métodos:**

Este estudo consistiu em 41 casos pós-COVID19 (Grupo 1) e 41 indivíduos saudáveis (Grupo 2). Apenas os olhos direitos dos participantes foram incluídos no estudo. A espessura da coroide foi medida usando tomografia de coerência óptica com profundidade de imagem aprimorada. Nos casos pós-COVID19, as medições foram realizadas dentro de 1 mês da doença. A espessura da coroide foi medida por dois oftalmologistas experientes nos quadrantes subfoveal, temporal e nasal, em sete pontos diferentes, a intervalos de 500 a 1500 µm da fóvea. Além disso, a espessura macular central e a espessura da camada de células ganglionares foram medidas com OCT e os dois Grupos foram comparados.

**Resultados:**

As espessuras coroidais foram estatisticamente mais espessas no Grupo 1 que no Grupo 2, com 500 µm no quadrante subfoveal, 500 Symbol (OTF)m no temporal e 1000 µm no nasal (p=0,011, p=0,043, p=0,009 e p=0,019, respectivamente). Embora outras medidas tenham se mostrado mais espessas no Grupo 1, elas não foram estatisticamente significativas (p>0,05). Também não houve diferenças significativas entre os Grupos quanto à espessura macular central e à espessura da camada de células ganglionares (p>0,05).

**Conclusão:**

A espessura da coroide mostrou-se aumentada em pacientes pós-COVID19. Isso pode estar relacionado à inflamação que faz parte da patogênese do COVID19.

## INTRODUCTION

The severe acute respiratory syndrome coronavirus (SARS-CoV-2), which appeared in China in 2019, had spread rapidly from person to person, causing a pandemic in 2020^(1)^, which the World Health Organization termed coronavirus disease 2019.

Given its multi-systemic characteristic, COVID-19 has been found to cause various symptoms^(2)^, such as fever, shortness of breath, joint pain, headache, diarrhea, and loss of smell^(3)^, which can range from mild to severe.

Autopsies performed in patients who died due to COVID-19 revealed not only pulmonary involvement but also extrapulmonary spread of SARS-CoV-2 and systemic vascular disease. The virus was detected in respiratory secretions, feces, urine, and sweat^(4,5)^, as well as tears using conjunctival swabs^(6)^.

Evidence has suggested hypercytokinemia to be one of the important pathophysiological mechanisms of COVID-19^(7)^. In particular, one study showed that the increase in interleukin (IL)-6 plays an important role in increasing other cytokines and endothelial damage^(8)^. Moreover, COVID-19-associated vascular inflammation has been found to cause endotheliitis, resulting in coagulopathy^(9)^.

The choroid consists of a dense choriocapillaris layer that has been considered the most prominent blood supply in the body. Studies have shown that diseases causing systemic inflammation to increase choroidal thickness^(10,11)^. Therefore, the current study aimed is to investigate the effects of SARS-CoV-2 infection on the choroid.

## METHODS

A prospective, cross-sectional, case-control study involving the right eyes of 41 post COVID-19 cases (Group 1) and 41 age-matched healthy subjects (Group 2) was performed at the Ophthalmology Department of Tokat Gaziosmanpaşa University.

Patients diagnosed with COVID-19 did not have severe symptoms that would require hospitalization. Positive individuals were placed in isolation, and their treatment was arranged at home. Measurements were made between 2 and 4 weeks after a diagnosis was established. None of the patients exhibited symptoms at the time of measurement. Patients in group 1 were questioned regarding their history with the use of antiviral, antiaggregant, and antithrombotic drugs for treatment of the disease. None of the patients positive for COVID-19 had a history of steroid use that could have affected choroidal thickness. All subjects underwent a full ophthalmic examination, anterior segment examination with slit lamp biomicroscopy and undilated fundus examination.

The exclusion criteria were previous ocular trauma, ocular surgery and any ocular disease, high myopia, and hyperopia. Patients with any systemic diseases, such as diabetes mellitus and systemic hypertension, as well as those using systemic or topical medication were excluded.

Our study was conducted in accordance with the Helsinki Criteria, the information was made over the file, without using personal data. Our study protocol was approved by the local ethics committee and from the Scientific Research Platform of the Turkish Ministry of Health.

### Imaging and image analysis

**Figure 1:**
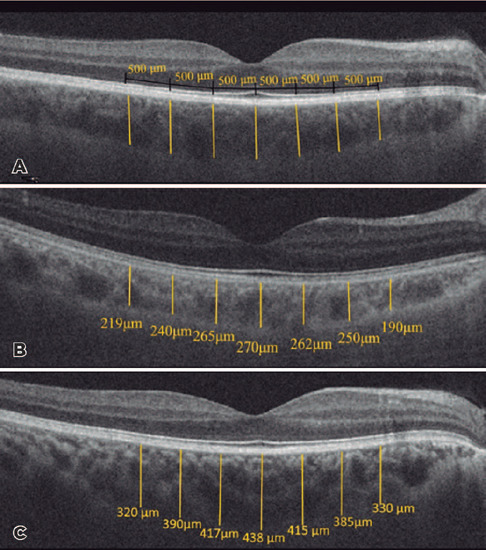
A, Segmentation of the choroid at 500-µm intervals. B and C, Choroidal thickness measurements in a healthy case and post-COVID-19 case, respectively.

All patients underwent OCT and EDI-OCT using the Cirrus Zeiss 5000 Zeiss. Choroidal thickness, central macular thickness, and ganglion cell thickness were compared between both groups. Choroidal thickness was measured by two experienced ophthalmologists (ŞGK and RK) who were blinded to the sessions using manual calipers of Cirrus HD-OCT software, after which the measurements were averaged for analysis. Measurements were made at 7 points: the subfovea and temporal and nasal quadrants at 500 µm intervals up to 1500 µm from the fovea. Choroidal thickness was measured from the outer edge of the hyper-reflective RPE to the inner sclera (Figure 1).

Statistical analysis was performed using SPSS 22.0. Variables were analyzed using visual and analytical methods (Kolmogorov-Smirnov/Shapiro-Wilk test) to determine whether or not they were normally distributed. Interobserver reproducibility was evaluated by analyzing the intraclass correlation coefficient (ICC). Descriptive analyses were presented using means and standard deviations for normally distributed variables. Student’s *t-*test was used to compare data between Groups 1 and 2, as well as according to sex and age. The chi-square test was used to compare data according to sex. A p value of <0.05 indicated statistical significance.

## RESULTS

Post-COVID-19 cases consisted of 20 (48.8%) men and 21 (51.2%) women, whereas healthy subjects consisted of 17 (41.5%) men and 24 (58.5%) women. Post-COVID-19 cases and healthy subjects had a mean age of 34.1 ± 8.3 (range 19 to 50) years and 30.7 ± 7.8 (range 20 to 47) years, respectively. No significant difference in age and sex were observed between the two groups (p=0.063 and 0.51, respectively) (Table 1).

**Table 1:** Demographic characteristics of the groups

	Post COVID-19 cases n=41	Healthy subjects n=41	p value
Age (mean ± SD)	34.1 ± 8.3	30.7 ± 7.8	0.51
Gender n (%)			
Men	20 (48.8)	17 (41.5)	0.063
Women	21(51.2)	24 (58.5)	

Group 1 had a thicker choroid compared to Group 2 at all measurement points. Mean subfoveal choroidal thickness was 370.60 ± 73.4 µm and 331.48 ± 62.1 µm in Groups 1 and 2, respectively, with a significant difference having been found between the two groups (p=0.011). Significant differences in the measurements made from the fovea at 500 µm nasal and temporal and 1000 µm nasal were observed between both groups (p=0.009, 0.043, and 0.019, respectively). However, no significant differences in the other measurements were observed (p>0.05). Interobserver measurements were performed at the 95% confidence interval (Table 2).

**Table 2:** Choroidal thickness measurements and intraclass correlation coeficient

Variables (Mean ± SD)	Group 1	Group 2	p value	ICC (95% Cl)
Sub-fovea choroidal thickness (µm)	370.60 ± 73.4	331.48 ± 62.1	**0.011** *	0.945 (0.907-0.967)
Choroidal thickness at 500 µm nasal to the fovea (µm)	348.04 ± 70.7	309.43 ± 58.7	**0.009** *	0.940 (0.892-0.966)
Choroidal thickness at 1000 µmnasal to the fovea(µm)	325.73 ± 67.1	291.24 ± 62.9	**0.019** *	0.947 (0.907-0.969)
Choroidal thickness at 1500 µm nasal to the fovea (µm)	295.12 ± 66.54	268.87 ± 59.2	0.63	0.933 (0.887-0.960)
Choroidal thickness at 500 µm temporal to the fovea (µm)	351.26 ± 73.8	320.26 ± 61.7	**0.043** *	0.968 (0.945-0.981)
Choroidal thickness at 1000 µm temporal to the fovea (µm)	330.70 ± 72.6	306.53 ± 62.7	0.11	0.855 (0.759-0.912)
Choroidal thickness at 1500 µm temporal to the fovea (µm)	315.68 ± 70.4	297.09 ± 63.3	0.21	0.824 (0.708-0.894)

*= Statistically significant p values.

ICC= Intraclass Correlation Coefficient.

Groups 1 and 2 had a central macular thickness of 249.2 ± 20.3 µm and 247.7 ± 18.6 µm (p=0.71) and ganglion cell layer thickness of 84.7 ± 4 µm and 83.4 ± 5 µm (p=0.26), respectively.

## DISCUSSION

To initiate infection in humans, SARS-CoV-2 needs to bind with angiotensin converting enzyme-2 (ACE-2)^(7)^, which is widely expressed in the heart, kidneys, gastrointestinal tract, and other humans tissues. This explains the wide variety of symptoms that occur with SARS-CoV-2 infection^(12)^.

ACE-2 expression has been observed in the cornea and conjunctival cells, making the ocular surface a potential target for SARS-CoV-2. However, protective mechanisms, such as lactoferrin and acetylsalicylic acid found in tears, can prevent SARS-CoV-2 from binding to such tissues.

A study investigating the presence of viruses in the retina found that 3 out of 14 eyes had SARS-CoV-2 viral RNA in the retina^(13)^.

Inflammation is one of the important pathophysiological mechanisms of COVID-19. As a defense mechanism against COVID-19, the body increases its expression of some cytokines (e.g., IL-1, IL-6, and vascular endothelial growth factor) and some chemokines (e.g., interferon gamma inducible protein, monocyte chemoattractant protein-1)^(14,15)^.

In the immune response to infection, the level of inflammatory mediators increases together with blood flow, thereby supporting post-infection vein dilatation. When the disease and inflammation are under control, a decrease in vein dilatation occurs^(16)^.

COVID-19 has been found to cause microvascular changes. Notably, a study by Invernizzi et al. that compared retinal findings between individuals exposed and unexposed to SARS-CoV-2 found that both mean arterial diameter and mean vein diameter were higher in those with COVID-19^(17)^.

Evidence has shown that the inflammatory state of SARS-CoV-2-infected patients is associated with vascular leakage^(18)^. Moreover, the immune response causes endothelial damage in the capillaries and increases coagulation, resulting in microvascular thrombosis^(19)^.

The choroid has been considered the tissue with the highest blood supply in the body, and the blood volume that reaches the eye is mostly found in the choriocapillaries^(20)^. Several studies have used OCT to evaluate choroidal thickness in cases of increased inflammation^(21,22)^. Cases with increased systemic inflammation, such as those with Vogt-Koyanagi-Harada and Behçet’s disease, have been reported to demonstrate increased choroidal thickness, which decreased with systemic anti-inflammatory therapy during follow-up^(23,24)^. We believe that patients with COVID-19 might have increased choroidal thickness due to systemic inflammation associated with COVID-19, which has been the premise of the current study. One limitation of this study is that measurements could only be carries out post COVID-19 due to the high contagiousness of the disease in the acute period. Choroidal thickness varies widely from person to person. The current study found that post-COVID-19 cases had significant greater choroidal thickness at the subfovea and 500 µm nasal of the fovea, suggesting that COVID-19 affects the subfoveal area the most.

In conclusion, post-COVID-19 cases had a significantly thicker choroid compared to healthy subjects, suggesting that inflammation associated with COVID-19 may affect the choroid.
